# Compressed Medicated Chewing Gum with Lysozyme Hydrochloride and Ascorbic Acid for Xerostomia Relief and Oral Health Support: Formulation Development, Optimization, *In Vitro* and *In Vivo* Evaluation

**DOI:** 10.3390/pharmaceutics18060700

**Published:** 2026-06-07

**Authors:** Yuliia Maslii, Nataliia Herbina, Olena Ruban, Jurga Bernatoniene

**Affiliations:** 1 Department of Drug Technology and Social Pharmacy, Faculty of Pharmacy, Medical Academy, Lithuanian University of Health Sciences, LT-50161 Kaunas, Lithuania; yuliia.maslii@lsmu.lt (Y.M.); nataliia.herbina@lsmu.lt (N.H.); 2 Department of Industrial Technology of Medicines and Cosmetics, National University of Pharmacy, 61002 Kharkiv, Ukraine; ruban@nuph.edu.ua; 3 Institute of Pharmaceutical Technologies, Faculty of Pharmacy, Medical Academy, Lithuanian University of Health Sciences, LT-50161 Kaunas, Lithuania

**Keywords:** xerostomia, medicated chewing gum, compression method, lysozyme hydrochloride, ascorbic acid, formulation, evaluation

## Abstract

**Background**: Existing therapies for xerostomia are primarily symptomatic, providing temporary mucosal hydration without addressing underlying pathological changes in the oral cavity. In this context, medicated chewing gums containing ascorbic acid and lysozyme hydrochloride offer a promising approach, combining antimicrobial, antioxidant, and trophic effects with physiological salivary stimulation and prolonged local delivery. **Methods**: For the development of compressed chewing gum formulation, the physicochemical (particle size distribution, moisture absorption capacity, and microscopic characteristics) and technological (flowability, angle of repose, bulk and tapped density, Carr’s index (CI), and Hausner ratio (HR)) properties of the active substances and their formulations with excipients were evaluated. Pharmacological activity was assessed in an atropine-induced xerostomia rat model. **Results**: The physical mixture of all components showed inferior flow properties compared with the formulation containing pre-granulated lysozyme hydrochloride, as evidenced by higher Carr’s index and Hausner ratio values (CI = 17, HR = 1.20 vs. CI = 13, HR = 1.14), indicating improved processability after pre-granulation. The effect of relative humidity during formulation was also assessed, with an optimal level of 40% required to ensure process stability due to the hygroscopic nature of the components. Based on these data, technological approaches ensuring processability were established, including wet pre-granulation of lysozyme hydrochloride and premixing of ascorbic acid to reduce oxidation risk. These approaches resulted in an optimized compression mass with excellent flowability (CI = 8, HR = 1.09), suitable for the preparation of medicated chewing gum. An optimal compression force (7 kN) ensured suitable rheological and textural properties, resulting in rapid and nearly complete release of the active ingredients from the medicated chewing gum, consistent with kinetic analysis. *In vivo* studies using an atropine-induced xerostomia rat model demonstrated that the combination of ascorbic acid and lysozyme hydrochloride significantly increased salivary secretion (2.17-fold vs. control pathology group) and reduced salivary gland mass coefficients (by 13–18% compared with the control pathology group and groups receiving individual active ingredients), alongside improvement of oxidative stress markers, including a reduction in TBA-reactants (by 51.6%) and an increase in catalase activity (by 51.0%). **Conclusions**: The developed medicated chewing gum showed favorable technological properties, efficient release of active ingredients, and anti-xerostomic activity *in vivo*, indicating its potential for xerostomia relief and oral health support.

## 1. Introduction

Xerostomia is a multifactorial pathological condition characterized by a subjective sensation of dryness in the mouth, usually associated with a reduction in saliva flow (hyposalivation) and/or a change in the qualitative composition of saliva [1,2]. Its prevalence in the general adult population is reported to range from approximately 5–46%, while in older adults it may reach 40–70%, and in patients receiving polypharmacy or oncological treatment it can exceed 70–80% [3,4,5]. The main causes include inflammatory dental disorders [6,7,8], medication-induced mouth dryness [9,10,11,12], systemic pathologies (endocrine, autoimmune and metabolic disorders) [13,14], as well as the effects of radiotherapy and chemotherapy [15,16], which are accompanied by damage to the secretory apparatus of the salivary glands. Other contributing factors include surgical procedures in the maxillofacial region [17], dehydration and disturbances in water and electrolyte balance [18], as well as functional and behavioural factors, including chronic stress [19], smoking [20], dietary disorders and reduced masticatory load [21]. These factors collectively disrupt the neurohumoral regulation of salivation and a reduce saliva production.

Saliva is a key component of oral homeostasis, performing protective, buffering, remineralizing and antimicrobial functions [22,23,24]. A reduction in saliva production leads to an imbalance between the processes of demineralization and remineralization of hard dental tissues, changes in the oral microbial flora, and a reduction in local immune defense. As a result, patients with xerostomia face a significantly increased risk of developing caries, inflammatory periodontal diseases, oral mucosal candidiasis, as well as erosive-ulcerative and traumatic lesions [25,26]. In addition, functional impairments are observed, including difficulties with chewing, swallowing and speech, which significantly reduce patients’ quality of life [27].

Modern approaches to the treatment of xerostomia include the use of substitutes (artificial saliva), pharmacological stimulants of salivary gland secretion (e.g., cholinergic agents), as well as topical moisturizers in the form of gels, sprays and solutions [28,29,30,31]. Despite the wide range of treatments available, their clinical efficacy is often limited. Saliva substitutes are characterized by short-acting effects and require repeated application throughout the day; systemic salivary stimulants may cause undesirable side effects and have restrictions on their use; topical formulations often have poor retention on the mucous membrane, which reduces the duration of their therapeutic effect [32]. A further drawback is poor patient compliance, due to the inconvenience of using certain dosage forms [33].

In this context, a key area of focus is the development and implementation of alternative dosage forms that both stimulate physiological salivation and provide sustained release of active ingredients in the oral cavity. One promising form is medicated chewing gum (MCG), which combines mechanical stimulation of the salivary glands with the ability to deliver active pharmaceutical ingredients (APIs) in a controlled manner. Its use helps to increase the volume and buffering capacity of saliva, improve the self-cleansing processes of the oral cavity and enhance remineralization potential [34,35,36]. Furthermore, this dosage form is characterized by its ease of use and high patient adherence to treatment, making it a promising option for the prevention and symptomatic treatment of xerostomia [35,36,37,38,39].

**Figure 1:**
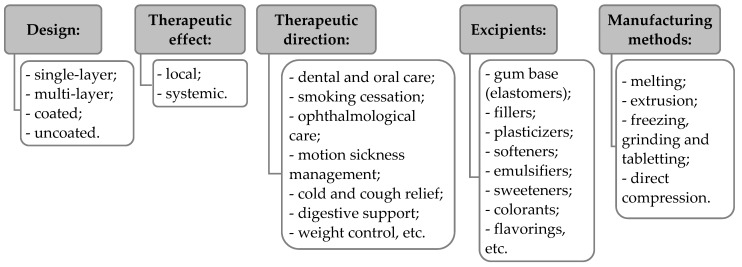
Characteristics and composition of MCG.

The characteristics of MCG as a dosage form and its main properties are shown in Figure 1.

The mechanism of MCG action in xerostomia is based on a combination of mechanical-reflex stimulation of salivation and the controlled release of APIs from a polymeric chewing matrix. During the initial stage of chewing, thermomechanical softening of the base occurs, accompanied by an increase in the diffusion mobility of the active components and the initiation of mass transfer processes. At the same time, reflex activation of the salivary glands occurs, which is critically important for patients with xerostomia, as it helps to increase saliva volume and partially restore its physicochemical properties. Further release of the active ingredients follows a diffusion-controlled mechanism, which is determined by the characteristics of the chewing base, the solubility of the active ingredients, and the intensity of chewing. The released components are distributed in saliva and ensure prolonged contact with the mucous membrane, thereby increasing local bioavailability and the efficacy of the pharmacological action. The therapeutic effect may include moisturization of the mucous membrane, normalization of the acid–base balance, modification of microbial biocenosis, and prevention of demineralization of hard dental tissues. Therefore, MCGs act as a combined delivery system, producing a synergistic effect through the combination of stimulation of physiological salivation and the controlled release of APIs, making them a promising dosage form for the prevention and symptomatic treatment of xerostomia [21,34,40,41].

In modern dentistry, symptomatic treatment of xerostomia involves the use of saliva-stimulating MCGs, which are sugar-free, xylitol-containing polymer systems, and in some cases, further enriched with organic acids or mineral components. The most common commercial products include DENTAID^®^ Xeros Chewing Gum (DENTAID, Barcelona, Spain), Vitis^®^ Xeros (DENTAID, Barcelona, Spain), Miradent^®^ Aquamed Chewing Gum (Hager & Werken GmbH & Co. KG, Duisburg, Germany), Spry^®^ Dental Defense Gum (Xlear Inc., American Fork, UT, USA), and Biotène^®^ Dry Mouth Gum (Haleon plc, Weybridge, Surrey, UK), which are primarily based on xylitol and are marketed as adjunctive therapies for xerostomia and caries prevention. The therapeutic effect of these products is due to a combination of masticatory stimulation and the local action of xylitol and auxiliary ingredients, which helps to increase saliva production and improve subjective oral comfort in patients with xerostomia. However, their effect on the pathogenic mechanisms of xerostomia remains limited, as the absence of antimicrobial and reparative action prevents them from fully addressing the inflammatory and trophic disorders of the oral mucosa associated with this condition [42,43,44,45].

In this context, there is an obvious need to develop a combined dosage form in the MCG form containing lysozyme hydrochloride and ascorbic acid, which would address not only reduced salivation but also the secondary effects of hyposalivation. Lysozyme hydrochloride, as a component of the innate immune defense of saliva, exerts bactericidal and immunomodulatory effects through the enzymatic destruction of the cell walls of microorganisms, which helps to reduce microbial colonization and alleviate the inflammatory changes in the mucous membrane characteristic of xerostomia [46,47,48,49,50]. Ascorbic acid, with its potent antioxidant properties and role in collagen synthesis, helps to reduce oxidative stress, improve microcirculation and restore the barrier function of the oral mucosa, as well as accelerate the regeneration processes that are impaired by chronic salivary deficiency [51,52,53,54,55].

Thus, the inclusion of these active ingredients in chewing gum will enable the therapeutic effect to be extended from a purely symptomatic level to one based on pathophysiological principles, which underscores the relevance and promise of this development.

According to the literature, MCGs can be produced using methods such as melting; freezing, grinding and tabletting; extrusion; and direct compression [35,56,57]. The most promising method in pharmaceutical technology is direct compression, which ensures high dosing accuracy, uniform distribution of active ingredients, compositional stability and the absence of thermal effects. Furthermore, this method allows for the incorporation of a significant amount of excipients and the application of protective or flavour coatings. An additional advantage is its compatibility with tableting equipment, making it cost-effective and technologically convenient for the production of MCGs [34,58].

The aim of this study is the pharmaceutical development, formulation optimization and *in vitro* and *in vivo* evaluation of compressed MCGs containing lysozyme hydrochloride and ascorbic acid as a dosage form for the relief of xerostomia symptoms and the maintenance of oral health.

## 2. Materials and Methods

### 2.1. Materials

APIs: lysozyme hydrochloride (Bouwhuis Enthoven B.V., Raalte, The Netherlands), ascorbic acid (Foodchem International Corporation, Shanghai, China).

Excipients: Health in Gum^®^ (HiG^®^) brand PWD-01 (Cafosa Gum SA, Barcelona, Spain), sucralose (Solo Sucralose-Non Micronised NF, VB Medicare PVT. Ltd., Hosur, India), Nat Apple Flavor Wonf (Kerry Inc., Kuala Lumpur, Malaysia), Apple FLV LQD FA-BO2980 (Kerry Inc., Kuala Lumpur, Malaysia), Syloid^®^ 244FP (Grace GmbH & Co., KG, Worms, Germany), Aerosil brand 380 (Evonik Resource Efficiency GmbH, Essen, Germany), Neusilin^®^ ULP2 (Fuji Chemical Industry Co., Ltd., Toyama, Japan), Magnesium stearate (S.D. Fine Chemicals Ltd., Mumbai, India), ethanol 96% (pharmaceutical grade, Fabric Vilniaus degtinė, Vilnius, Lithuania).

Analytical Reagents: Lysozyme hydrochloride JP Reference Standard was obtained from Pharmaceuticals and Medical Devices Agency (PMRJ, Tokyo, Japan). A *Micrococcus luteus* strain was obtained from the American Type Culture Collection (ATCC, Manassas, VA, USA) and used for preparation of the bacterial suspension. Potassium iodide, soluble starch, hydrochloric acid, thiobarbituric acid, trichloroacetic acid, ammonium molybdate, hydrogen peroxide (30% solution), and thiopental sodium were purchased from Sigma-Aldrich (St. Louis, MO, USA). Phosphate-buffered saline (PBS, pH 7.4) was prepared using analytical-grade reagents and purified water. All reagents were of analytical grade and used as received.

### 2.2. Granule Preparation

Granules were prepared with lysozyme hydrochloride (1.0%) as the active component, together with flavour enhancers, including sucralose (0.15%) and a “Green Apple” flavouring powder (2.0%). The components were blended in a mixer to obtain a homogeneous powder mixture and then moistened with 96% ethanol as a granulating liquid, which was added gradually until a wet mass suitable for granulation was formed. The resulting wet mass was passed through a 2.0 mm sieve to form granules. The obtained granules were dried at room temperature until constant mass and subsequently calibrated through a 1.0 mm sieve to ensure uniform particle size distribution [59,60].

### 2.3. MCG Preparation

The obtained granules of lysozyme hydrochloride, ascorbic acid (2.0%), Health in Gum^®^ PWD 01 (91.75%), and Syloid^®^ FP244 (1.0%) were blended together to achieve a uniform mixture. To ensure the “Green Apple” flavour (0.6%) was distributed evenly, the aroma was sprayed onto the Syloid^®^ FP244 and mixed thoroughly until a free-flowing dry blend was obtained. Magnesium stearate (1.5%) was then added during the final blending stage to prevent the mass from adhering to the punches during compression.

The final blends were then compressed using a laboratory single-punch tablet press (HTM-01E, Mariupol Plant of Technological Equipment, Mariupol, Ukraine) equipped with a force-measuring device, to produce round, flat-faced medicated chewing gums with a mass of 1000 mg and a diameter of 13 mm.

### 2.4. Characterization of Powders, Granules, and Blends

#### 2.4.1. Flowability and Angle of Repose Determination

Flowability was determined by measuring the time required for powders (granulates) to flow through a funnel using a laboratory device (model VP-12A, MZTO, Melitopol, Ukraine). The angle of repose was measured using a protractor scale and calculated based on tan(α). All measurements were performed in triplicate for each sample.

#### 2.4.2. Bulk and Tapped Density Measurement

Bulk density, tapped density, Compressibility index (Carr’s index), and Hausner Ratio were determined using SOTAX TD1 Tap Density Tester (SOTAX, Westborough, MA, USA) using a 250 mL graduated cylinder. The unsettled apparent volume (V_0_) was recorded, and bulk density was calculated as m/V_0_. Tapped density was measured after 10, 500, and 1250 taps, recording the corresponding volumes (V_10_, V_500_, V_1250_). The final tapped volume (V_f_) was used to calculate tapped density as m/V_f_. The flow properties of the powder (granules, blends) were assessed based on Carr’s index and Hausner ratio values. All measurements were performed in triplicate for each sample.

#### 2.4.3. Microscopic Analysis

Optical microscopy was performed using a Konus Academy laboratory microscope (Konus, Verona, Italy) equipped with a ScopeTek camera (ScopeTek, Hangzhou, China). Images were acquired in reflected light and processed using Scope Photo software (version 3.0.12.498).

#### 2.4.4. Moisture Absorption Capacity

Moisture uptake was evaluated using samples weighing 1.0 g. The study was conducted at room temperature under controlled relative humidity (RH) conditions of 40%, 60%, and 75% for 24 h using a desiccator. The samples were placed in open weighing dishes, and their mass increase was determined gravimetrically after exposure. Moisture uptake was calculated as the percentage increase in mass relative to the initial sample weight. All measurements were performed in triplicate for each sample.

#### 2.4.5. Sieve Analysis

The granulometric composition was determined by analytical sieving using a vibration sieve shaker (VEB MLW Labortechnik Ilmenau, Ilmenau, Germany) equipped with a set of SLM-200 laboratory sieves with mesh sizes of 1.0, 0.7, 0.5, 0.355, 0.25, and 0.09 mm. A 100.0 g sample was used for the analysis. The sieving was performed until a constant mass distribution on the sieves was achieved. All measurements were performed in triplicate.

### 2.5. MCGs Evaluation

#### 2.5.1. Geometric Parameters

An Electronic Digital Caliper DIN 862 (Vogel Germany GmbH & Co. KG, Kevelaer, Germany) was used to determine the diameter and thickness of the MCGs. Measurements were performed on ten individual dosage units.

#### 2.5.2. Mechanical Resistance

The mechanical resistance of the MCGs was evaluated according to the pharmacopoeial methods ‘Resistance to Crushing of Tablets’ (Ph. Eur. 9.0, Chapter 2.9.8) [61] and ‘Friability of Uncoated Tablets’ (Ph. Eur. 9.0, Chapter 2.9.7) [62]. Compressed MCG crushing strength was determined using a Monsanto Hardness Tester (Campbell Electronics, Mumbai, India), whereas friability testing was carried out with a PTF 20E Friability Apparatus (Pharma Test, Hainburg, Germany). Ten dosage units were tested for friability at a drum rotation speed of (25 ± 1) rpm for 100 rotations (approximately 4 min).

#### 2.5.3. Texture Profile Analysis

The texture properties of MCGs were evaluated using a penetration test performed on a TA.XT.plus texture analyzer (Stable Micro Systems Ltd., Godalming, Surrey, UK). A stainless-steel needle probe (P/2N, 2 mm thickness) was used to assess sample deformation. Each gum sample was positioned centrally beneath the probe, which was driven into the material at a constant load of 5 kg and a speed of 2 mm/s to a penetration depth of 3 mm. Two primary parameters were recorded: hardness, defined as the maximum force required for the probe to overcome the mechanical resistance of the sample during penetration, and adhesiveness, defined as the force required to overcome the sticking (adhesive) interactions between the probe surface and the sample. All measurements were conducted at room temperature (25 ± 2 °C). Three independent samples were analyzed, with each measurement performed in triplicate.

#### 2.5.4. *In Vitro* Drug Release Study

Drug release from MCGs was evaluated using the dissolution method in accordance with the pharmacopoeial test “Dissolution Test for Medicated Chewing Gums” (Ph. Eur. 9.0, Chapter 2.9.25) [63], which simulates the chewing process (Erweka GmbH, Langen, Germany). The dissolution conditions were as follows: 60 chewing cycles per minute; a distance of 1.4 mm between the chewing surfaces; 20.0 mL of phosphate buffer R2 solution (pH 6.0) as the dissolution medium; a temperature of 37 ± 1 °C; and sampling time points at 5, 10, 15, 20, and 30 min. At each time point, 2.0 mL of the sample was withdrawn and replaced with an equal volume of pre-heated phosphate buffer R2 solution. The test was performed on six MCGs from each batch, with different compression forces applied (5 kN, 7 kN, 10 kN, and 15 kN).

The lysozyme hydrochloride assay was performed using a Specord 200 spectrophotometer (Analytik Jena AG, Jena, Austria) in accordance with the Japanese Pharmacopoeia (JP XVII) monograph for lysozyme hydrochloride [64]. To prepare the test solution, a 1 mL aliquot of the sample was introduced into a volumetric flask and brought to volume with phosphate buffer (pH 6.2), resulting in an estimated lysozyme hydrochloride concentration of approximately 0.01 mg/mL. A 10 mL suspension of *Micrococcus luteus* in the same buffer, adjusted to an absorbance of approximately 0.65 at 640 nm, was then added. The absorbance at 640 nm was subsequently recorded. Quantification was performed using two standard solutions prepared from the Lysozyme JP Reference Standard at concentrations of 0.01 and 0.005 mg/mL.

The ascorbic acid assay was performed by redox iodometric titration in accordance with the Ph. Eur. [65]. A 1 mL aliquot of the sample was mixed with 10 mL of 0.1 M hydrochloric acid, 0.5 mL of freshly prepared potassium iodide solution (10 g/L), and 2 mL of starch solution. The resulting mixture was titrated with 0.0167 M potassium iodate until the appearance of a violet-blue endpoint.

Drug release data were fitted to zero-order, first-order, Higuchi, and Korsmeyer–Peppas models. The corresponding equations are presented in Table 1. Model fitting was performed using linear/nonlinear regression, and the goodness of fit was evaluated using the coefficient of determination (R^2^).

### 2.6. In Vivo Study of Atropine-Induced Xerostomia in Rats

The study was conducted on 40 healthy outbred female rats aged 2.5–3 months, randomly assigned to experimental groups with minimized differences in body weight. The sample size (*n* = 8 per group) was selected based on previous studies using similar rat models of salivary gland dysfunction, in which small experimental groups are commonly employed, together with expected biological variability and Festing and Altman recommendations for experimental design [66]. The number of animals was selected to ensure adequate statistical sensitivity while minimizing animal use in line with the 3R principles.

Animals were housed in the vivarium of the Educational and Scientific Institute of Applied Pharmacy, National University of Pharmacy (Kharkiv, Ukraine), under a 12:12 h light/dark cycle at 22–24 °C, with food and water ad libitum. All procedures complied with EU Directives 86/609/EEC and 2010/63/EU [67,68] and were approved by the Bioethics Commission of the National University of Pharmacy (Approval No. 13 of 13 March 2024).

Animals were randomly divided into five groups (*n* = 8 per group):

- Intact control (IC): no induced pathology; solvent irrigation.
- Control pathology (CP): xerostomia induced; no treatment.
- Group CP + AsA: xerostomia induced; ascorbic acid treatment.
- Group CP + LH: xerostomia induced; lysozyme hydrochloride treatment.
- Group CP + AsA + LH: xerostomia induced; combined ascorbic acid and lysozyme hydrochloride treatment.

Experimental xerostomia was induced by daily oral administration of 0.01% atropine sulfate (0.05 mL) for 14 days under fasting conditions [69,70]. During this period, the oral cavity was irrigated once daily with test solutions or solvent (according to group allocation) 2 h after atropine administration.

Doses of active substances were extrapolated from clinical daily doses using interspecies scaling based on body weight and surface area. Thus, as an experimental analogue of chewing gum, a solution of lysozyme hydrochloride and ascorbic acid was administered at 0.004 g/kg and 0.008 g/kg, respectively. Reference groups received the corresponding monotherapies at equivalent doses.

After 2 weeks of treatment, salivation was assessed. Saliva was collected over 30 min under thiopental anesthesia (40 mg/kg) using a microcapillary tube, and the spontaneous salivation rate was expressed as mL/min. After measurements, animals were euthanized by CO_2_-induced hypoxia, and the parotid and submandibular glands were excised.

Submandibular salivary gland tissues were homogenized (10% *w*/*v*) in PBS-buffer [71] and the supernatant was used to assess pro-/antioxidant parameters. The content of thiobarbituric acid (TBA) reactants and catalase activity were measured as markers of oxidative status using standard methods [72]. TBA reactants levels were determined by reaction with thiobarbituric acid after protein precipitation with trichloroacetic acid, with absorbance measured at 532 nm. Catalase activity was assessed by a conventional method based on the formation of a colored complex between hydrogen peroxide and ammonium molybdate, measured at 410 nm.

### 2.7. Statistical Analysis

Statistical analysis was performed using Microsoft Office 365 Excel 2016 (Redmond, WA, USA) and IBM SPSS Statistics 22 (IBM Corp., Armonk, NY, USA). All experiments were conducted in triplicate (*n* = 3). The results of physicochemical, technological, and pharmacological studies were expressed as mean ± standard deviation (SD). Statistical comparisons between experimental groups and samples were performed using one-way analysis of variance (ANOVA). For pharmacological studies multiple comparisons between groups were additionally evaluated using Tukey’s honestly significant difference (HSD) post hoc test. Differences were considered statistically significant at *p* < 0.05 [73].

## 3. Results and Discussion

In addition to the APIs and the chewable base, every compressed MCG contains excipients that guarantee the required organoleptic, physicochemical and functional properties of the dosage form, ensuring stability and ease of use. These components include sweeteners, flavourings, thickening agents and lubricants, each of which serves a specific technological and consumer purpose. Table 2 presents the main components of the compressed MCGs under development, their functional purpose and the critical parameters affecting the properties and efficacy of the chewable dosage form; these ingredients were selected on the basis of data from the scientific literature and pharmaceutical practice in the development of similar delivery systems [37,56,74].

In the pharmaceutical development of any medicinal product, it is important to investigate the physicochemical and technological properties of its constituents, as these directly influence the choice of production method of the dosage form, the way for introducing the APIs into the formulation and their uniform distribution, as well as the process conditions and the product’s consumer qualities [75,76,77].

In particular, the flowability of powders is one of the key characteristics that determines the course of technological processes, including dosing, mixing and the formulation of dosage forms. An analysis of the technological characteristics of the substances presented in Table 3 indicates that HiG^®^ PWD-01 exhibits good flowability, as evidenced by low values for the Hausner coefficient and the Carr’s index. This will ensure uniform dosing, effective mixing and stable shaping of the powder mass. Ascorbic acid exhibits fair flowability and a low tendency to form lumps, which makes it suitable for use in multi-component mixtures. At the same time, lysozyme hydrochloride, based on its high angle of repose value, Hausner ratio and Carr’s index, is characterized by passable flowability, indicating a tendency to clump and potential difficulties during dosing and tableting. Thus, HiG^®^ PWD-01 and ascorbic acid have sufficient flowability for standard processing operations, whereas lysozyme hydrochloride may require additional measures to improve its flowability.

**Figure 2:**
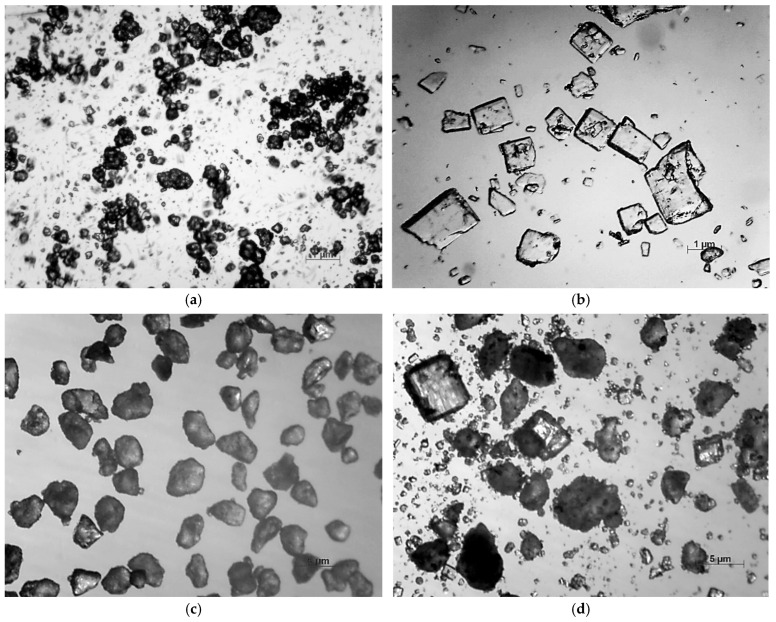
Crystallographic analysis of powders: (**a**) LH; (**b**) AsA; (**c**) HiG^®^ PWD-01; (**d**) mixture of APIs with HiG^®^ PWD-01.

Furthermore, the shape and size of the powder particles determine its flowability, which has a critical impact on the uniformity of dosing and distribution within the mass during pressing [78]. In this regard, we carried out a crystallographic analysis of APIs, the HiG^®^ PWD-01 chewable base and their mixture in order to evaluate their properties with a view to optimizing the tableting process. The results are shown in Figure 2. The analysis established that the mixture of APIs with the HiG^®^ PWD-01 composition constitutes a polydisperse system containing particles of varying shapes, which prevents the formation of a homogeneous mass suitable for direct compression and leads to segregation during the MCG production process.

**Figure 3:**
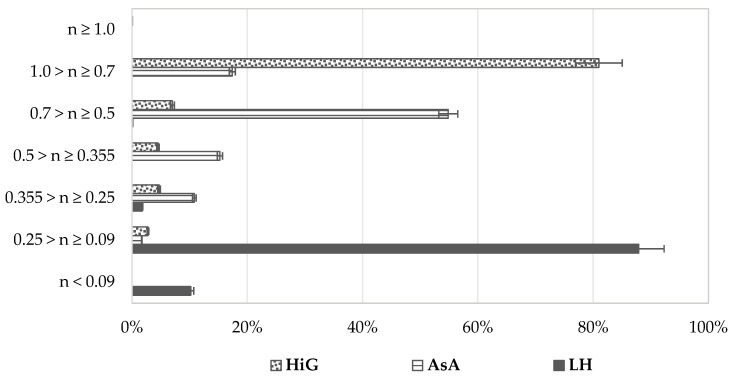
Particle size distribution of the APIs and HiG^®^ PWD-01: n—particle size of the substances, mm (*n* = 3).

The polydispersity of the system is indicated by the results of sieve analysis: the main particle size fraction of lysozyme hydrochloride is within the range of 0.09–0.25 mm, that of ascorbic acid is 0.5–0.7 mm, and the HiG^®^ PWD-01 chewable base—0.7–1.0 mm (Figure 3). This suggests that a more homogeneous system may be achieved by mixing the chewable base with ascorbic acid, as the particle sizes of this API are close to those of the HiG^®^ composition. At the same time, mixing the base with both APIs simultaneously may not ensure the formation of a homogeneous system or the uniform distribution of lysozyme hydrochloride within the chewing gum mass.

**Figure 4:**
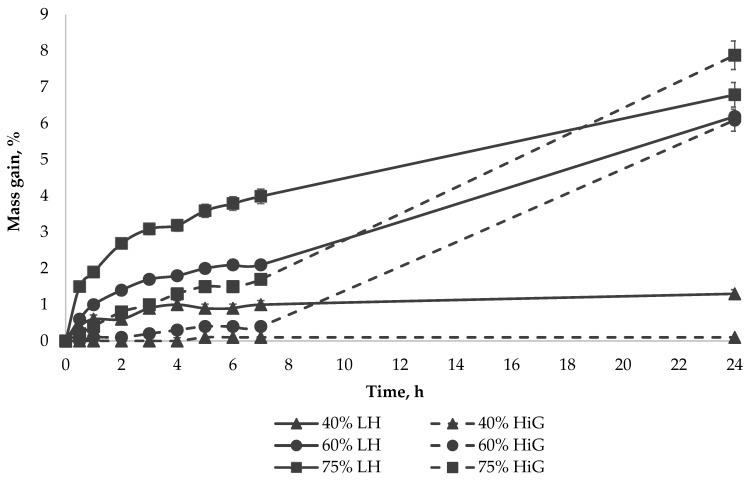
Moisture absorption of LH and the HiG^®^ PWD-01 chewable base at different RH levels. Data are expressed as mean ± SD (*n* = 3). Statistical analysis was performed using one-way ANOVA, and differences were considered significant at *p* < 0.05.

It is well known that the hygroscopicity of powders has a negative impact on the quality of the compressed product, both during processing and during storage, and may also reduce the bioavailability of the active ingredients [78]. The moisture-absorption capacity of the API and the chewable base was therefore investigated over a 24 h period at relative humidity (RH) levels of 40%, 60%, and 75%, corresponding to the physiologically optimal humidity range (40–60%) and the maximum permissible value (75%) in the manufacturing processes of pharmaceutical companies. According to the results presented in Figure 4, the mass of the ascorbic acid samples remained unchanged at all investigated relative humidity levels, confirming the non-hygroscopic nature of this API. In contrast, lysozyme hydrochloride exhibited rapid moisture uptake under all humidity conditions, with moisture absorption increasing significantly during the initial storage period and approaching equilibrium after approximately 6–7 h. One-way ANOVA demonstrated a significant effect of relative humidity and storage time on the moisture absorption behaviour of both lysozyme hydrochloride and HiG^®^ PWD-01 (*p* < 0.05). For lysozyme hydrochloride, moisture uptake increased significantly with increasing RH, reaching 1.3%, 6.19%, and 6.79% mass gain after 24 h at 40%, 60%, and 75% RH, respectively. The most pronounced increase was observed during the first hours of exposure. In contrast, HiG^®^ PWD-01 showed negligible moisture uptake at 40% RH throughout the study period. At 60% and 75% RH, a delayed sorption pattern was observed, characterized by relatively low initial moisture absorption followed by a marked increase after prolonged exposure. After 24 h, the mass gain reached 6.09% and 7.88% at 60% and 75% RH, respectively. The moisture uptake at 75% RH was significantly higher than that observed at 40% RH (*p* < 0.05). Comparison of the sorption profiles indicates distinct moisture absorption mechanisms. Lysozyme hydrochloride was characterized by a significantly higher initial adsorption rate, whereas HiG^®^ PWD-01 remained relatively stable under low-humidity conditions and became increasingly hygroscopic only at elevated RH values. These differences are important for powder compaction processes, as rapid moisture absorption by lysozyme hydrochloride may adversely affect powder flowability and distribution uniformity, whereas excessive moisture uptake by HiG^®^ PWD-01 at high humidity may promote particle agglomeration and compromise the quality of compressed products. Overall, statistical analysis confirmed that environmental humidity and exposure time significantly affected moisture absorption behaviour (one-way ANOVA, *p* < 0.05), highlighting the importance of humidity control and, where appropriate, the incorporation of moisture-absorbing excipients to maintain the technological properties of the powder mixtures.

Therefore, for direct compression of MCG, it is necessary to ensure the homogeneity and uniform distribution of the APIs within the mixture. However, due to the polydispersity of the system and the unsatisfactory physicochemical and technological properties of lysozyme hydrochloride, this requirement cannot be met without prior granulation of the substance. The most effective method for converting powders into granules is wet granulation, which involves wetting the powdered mixture with a granulating liquid, followed by granulation of the wet mass and drying [79]. In order to eliminate any adverse effects of excipients on oral tissues, it was decided to granulate lysozyme hydrochloride using 96% ethanol, which enables the rapid production of high-quality granules.

To optimize the formulation, it is advisable to incorporate ascorbic acid into the mixture by premixing it with lysozyme hydrochloride granules and a chewable base, given its satisfactory processing characteristics and in order to prevent oxidation upon contact with the humectant.

**Figure 5:**
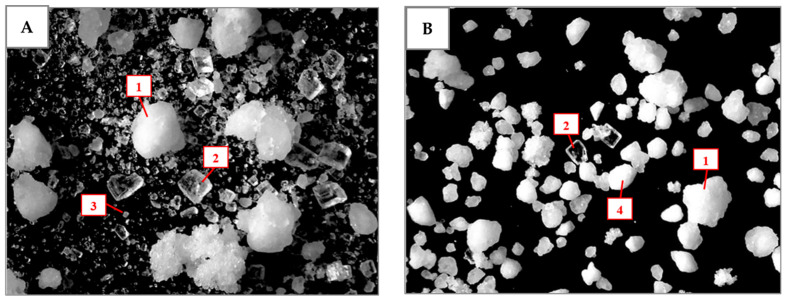
Comparative crystallographic analysis of MCG mixtures (magnified 3.3 times): (**A**) mass I; (**B**) mass II; 1—HiG^®^ PWD-01 particles, 2—AsA particles, 3—LH and other excipients particles, 4—granules obtained from LH, sucralose and flavouring.

**Figure 6:**
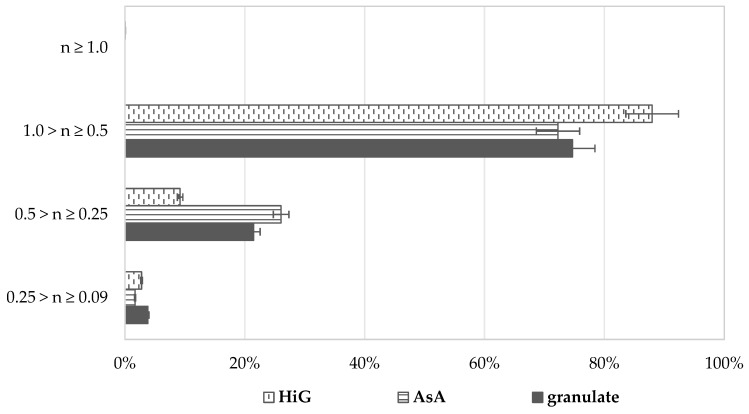
Particle size distribution of compression mass II: n—particle size of the substances, mm. Data are presented as mean ± SD (*n* = 3).

Thus, compared with the physical mixture (mass I), pre-granulation of lysozyme hydrochloride (mass II) resulted in a more uniform distribution of the APIs within the mixture, as confirmed by microscopic analysis (Figure 5), while sieve analysis (Figure 6) demonstrated a narrow and homogeneous particle size distribution within mass II. The particle size distribution was strongly dominated by the 0.5–1.0 mm fraction, which accounted for 72.26–87.97% depending on the formulation, while the fraction of particles below 0.25 mm did not exceed 3.83%, and particles larger than 1.0 mm were absent or negligible (<0.1%).

Technological studies conducted on the resulting mass for pressing II also showed better results compared to mass I, namely good flowability as opposed to fair (Table 4).

**Figure 7:**
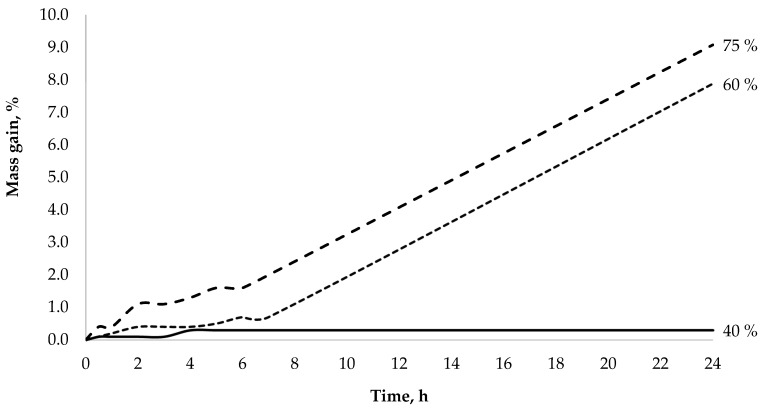
Moisture absorption of the mass for pressing II.

However, the moisture absorption capacity of the formed mass for pressing II did not decrease; on the contrary, it increased to 7.88% and 9.08% at 60% and 75% RH levels respectively (Figure 7), which is associated with the presence in the mixture of hygroscopic components such as lysozyme hydrochloride and a chewable base. At the same time, the moisture absorption of the mass at 40% RH remained at approximately the same level—0.30% throughout the 24 h observation period. This confirms previous findings regarding the necessity of adding a moisture-absorbing agent to the formulation or ensuring appropriate conditions during the production of MCG, namely an ambient 40% RH.

As recommended by the company “Cafosa” (Sant Cugat del Vallès, Barcelona, Spain), to enhance the flavour profile of MCG, in addition to an intense sweetener and a powdered flavouring agent, the formulation should include a water-insoluble flavouring agent, which provides a prolonged flavour effect and improves the plasticity of the mixture [45,80]. To ensure a harmonious blend of flavours, the flavouring agent in the MCG had the same flavour as the powdered additive—“Green Apple”.

The presence of an oil-based aroma flavouring agent in the MCG formulation necessitates the selection of a carrier capable of ensuring its uniform distribution within the pressing mass. Furthermore, previous studies have demonstrated the need to incorporate a moisture-absorbent agent due to the impact of moisture on the technical and consumer properties of the chewing gum. To this purpose, adsorbents were selected that enable the conversion of liquid APIs into powders with good flow properties, whilst also acting as a moisture-absorbing agent: Aerosil grade 380, Syloid^®^ 244FP [81,82,83] and Neusilin^®^ ULP2 [84,85].

The aroma flavouring solution was sprayed onto the chosen adsorbents and mixed until a homogeneous dry mixture was formed. Their selection was based on the degree of adsorption, the quantitative content in a single MCG (aroma:adsorbent ratio—1:2) and the ability to distribute the flavoring agent uniformly within the mixture, which, accordingly, will have a significant impact on the technological properties of the mass for pressing.

**Figure 8:**
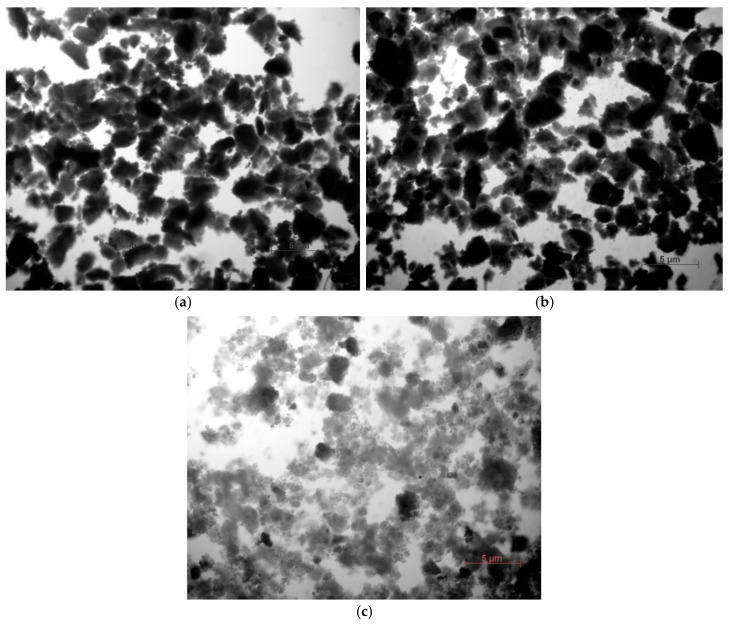
Microscopic analysis of adsorbent mixtures containing an aroma flavour: (**a**) Syloid^®^ 244FP; (**b**) Neusilin^®^ ULP2; (**c**) aerosil 380.

Microscopic analysis (Figure 8) revealed that all investigated adsorbents rapidly absorbed the flavouring solution, forming a dry, powdery mass. The flavouring mixture containing Syloid^®^ 244FP was characterized by a uniform distribution of particles within the field of view and consistent colour and particle size (1.5–2.5 µm), whereas the system containing Neusilin^®^ ULP2 contained particles of varying sizes (1.0–4.0 µm). The use of aerosil 380 was accompanied by uneven colour changes and the formation of agglomerates measuring 0.5–2.5 µm, which could lead to an uneven distribution of the correctant within the pressable mass. In view of these characteristics, aerosil 380 was excluded from further studies.

The system with Syloid^®^ 244FP exhibited the highest moisture absorption capacity. When added at 1.0% of the adsorbent into the pressing mass for 24 h at 75% RH, the mass gain was 7.78% for Syloid^®^ and 8.08% for Neusilin^®^, which reduced the initial moisture content of the mass by 1.3% and 1.0% respectively. At 60% RH, the mass gain was lower than the initial value (6.79% for Syloid^®^ and 7.49% for Neusilin^®^). No statistically significant changes in mass were observed at 40% RH.

According to the literature, the adsorbents under investigation are also capable of influencing the flow properties of powders, acting as glidants in the production of solid dosage forms [86,87]. Consequently, in order to make a final selection of a suitable moisture-absorbing agent and carrier for the oil-based flavouring in the composition of compressed MCGs, we investigated the key technological properties of the mixture containing these substances at a concentration of 1.0%. The data presented in Table 5 show that mixtures with Syloid^®^ 244FP and Neusilin^®^ ULP2 demonstrate good flowability and compressibility characteristics. According to the European Pharmacopoeia specifications, the flow properties for both mixtures are classified as “good”, which is supported by the values for the angle of repose, Carr’s index, and Hausner ratio. Statistical evaluation using Student’s *t*-test revealed that the powder mass containing Syloid^®^ 244FP exhibited a statistically significant (*p* < 0.05) improvement in flowability (6.13 ± 0.21 s/100 g) compared to the Neusilin^®^ ULP2 batch (6.59 ± 0.09 s/100 g). However, no significant differences (*p* > 0.05) were observed between the two adsorbents across the remaining technological indicators, indicating that both excipients provide comparable and highly acceptable processing properties for tableting. Therefore, taking into account the results of all the studies—including the enhanced powder rheology, superior adsorbent capacity, and moisture-regulating properties—Syloid^®^ 244FP was selected as the optimal carrier.

**Figure 9:**
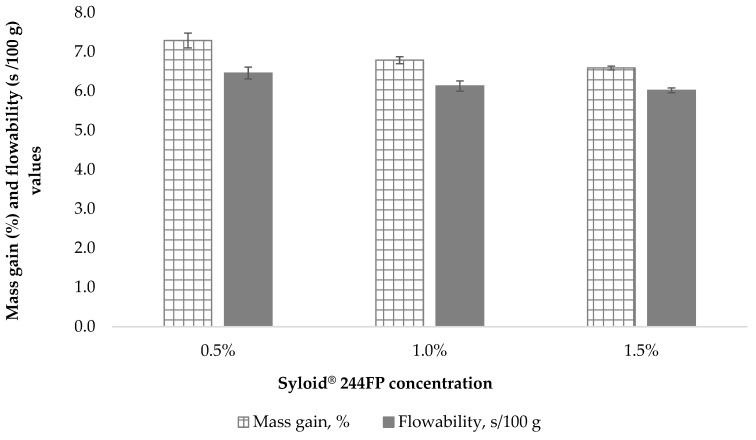
Effect of Syloid^®^ 244FP concentration on the moisture-absorbing capacity and flowability of the mass for pressing. Data are presented as mean ± SD, *n* = 3.

In order to determine the optimal concentration of the selected adsorbent, Syloid^®^ 244FP was incorporated into the mass for pressing at concentrations of 0.5%, 1.0%, and 1.5%. The effect of the adsorbent concentration on the moisture absorption capacity and flowability of the mass was investigated. The moisture-absorbing capacity was assessed over a 24 h period at 60% RH (Figure 9). Data are presented as mean ± SD (*n* = 3), and statistical analysis was performed using one-way ANOVA. Increasing the concentration of Syloid^®^ 244FP improved the investigated parameters; however, no statistically significant difference was observed between the 1.0% and 1.5% concentrations. Consequently, 1.0% was selected as the optimal concentration in the MCG formulation.

The main disadvantage of obtaining this dosage form by compression is the tendency of MCG to adhere to the punches of the tablet press, which is due to the adhesive nature of the chewable gum base, as the main dominant component of the preparation. The literature recommends reducing the compression speed, cooling the mass and the compression tools to 10–18 °C, coating the punches with Teflon, or using lubricants to overcome this problem [88]. Lubricants are usually added during the final stages of mixing the components prior to compression. Magnesium stearate is widely used in the manufacture of solid dosage forms as an effective and stable anti-friction agent with a high melting point (117–150 °C) and low cost [89]. In our studies, a high-quality product was only obtained at a magnesium stearate concentration of 1.5%, whereas lower concentrations caused the gum to stick to the upper punch.

As shown in Table 6, after adding 1.5% magnesium stearate, the flowability of the mixture is rated as excellent, which will ensure uniform dosing and the production of high-quality compressed MCGs.

Given the method used to produce MCG, one of the key parameters affecting the quality of the medicinal product is the optimal compression pressure. Therefore, in order to determine the optimal compression force, we decided to investigate its effect on the organoleptic, mechanical, textural and biopharmaceutical characteristics of MCG.

Chewing gum samples weighing 1.0 g and with a diameter of 13 mm were produced using an HTM-01E single-punch tablet press with compression forces of 5, 7, 10, and 15 kN [58,90,91]. The geometric parameters and strength characteristics of the four MCG batches compressed at different force values are presented in Table 7.

As shown in the results in Table 7, the compression force affected the tested parameters of the chewing gum. One-way ANOVA confirmed that an increase in pressure during production led to a statistically significant (*p* < 0.05) reduction in thickness and a concurrent increase in the MCG’s resistance to mechanical stress across all studied batches. However, even the batch of chewing gum produced with the lowest compression force fully met the Ph. Eur. requirements for friability (Chapter 2.9.7) [62]. It should be noted that the strength test did not result in the gum being crushed, but only in its deformation, which is evidently due to the flexible and elastic nature of the MCG base. Unlike traditional tablets, compressed MCG is designed to be chewed, which results in a different mechanical behaviour. Standard methods for determining crushing resistance are inappropriate here, as the samples undergo elastic-plastic deformation rather than breaking. To objectively assess this characteristic, instrumental texture analysis was also used [91,92,93]. The texture profile of the gum was investigated by conducting a penetration test using the TA.XT.plus texture analyzer. According to the results shown in Table 7, a statistically significant upward trend (*p* < 0.05) was observed for both texture parameters, allowing the MCG batches to be ranked in the following order based on hardness and adhesion: 15 kN > 10 kN > 7 kN > 5 kN. In other words, as the compression force increases, the hardness of the gum and its adhesive capacity increase, which may lead to difficulties during chewing and sticking to the teeth and, consequently, to a deterioration in the product’s consumer characteristics.

**Figure 10:**
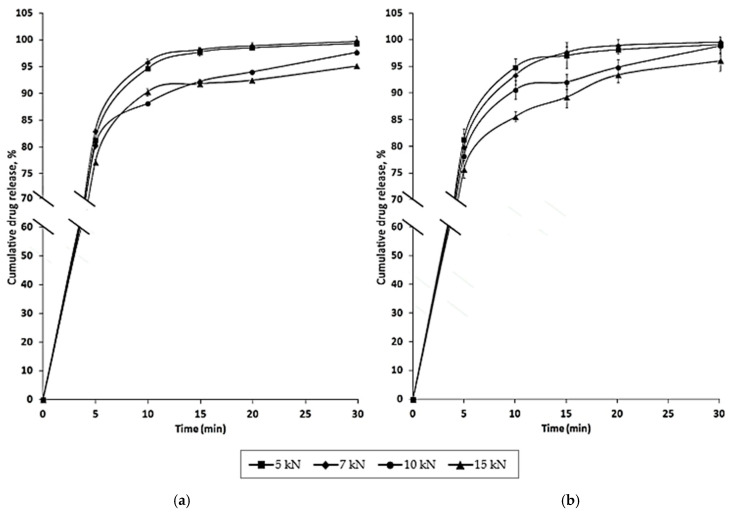
Cumulative release of LH (**a**) and AsA (**b**) from four MCGs batches compressed at different compression forces (values are expressed as average ± SD, *n* = 6).

The release profile of conventional tablets depends significantly on the hardness of the product and the solubility of the API [88,94,95,96]. Unlike conventional tablets, MCGs offer the advantage of being chewable, which promotes a more effective release of active ingredients. Consequently, even substances that are highly soluble in water, such as lysozyme hydrochloride and ascorbic acid, are released in the oral cavity not only by diffusion but primarily through mechanical stimulation. In this context, the effect of compression force on the kinetics of API release was also investigated (Figure 10).

Analysis of the kinetics of API release from MCG revealed a two-phase release profile for both active components. In the initial stage, the mechanical action of chewing leads to the breakdown of the matrix into fragments, increasing the surface area and accelerating the release of soluble components. In the subsequent stage, release is controlled primarily by diffusion through the compacted and less porous structure of the matrix. Thus, approximately 80% of the active substances had been released into the solution within just 5 min for all samples studied, indicating a pronounced initial phase of burst release. One-way ANOVA confirmed that the compression force exerted a statistically significant effect on the drug release rate during the dissolution process (*p* < 0.05). After 10 min, the average percentage of ascorbic acid released was 94.87% for 5 kN and decreased to 85.58% at 15 kN (the difference between these groups was statistically significant, *p* < 0.05), while a similar significant trend was observed for LH release. After 30 min, the differences between the batches decreased to 98.31% and 95.11% for AsA, respectively, becoming statistically non-significant (*p* > 0.05). Therefore, MCGs obtained at lower compression forces will ensure a more complete and statistically faster release of both APIs.

An analysis of the kinetic parameters presented in Table 8 revealed a similar trend: as pressure increased, the initial rate *k*_0_ decreased from 3.00 to 2.00%/min, the rate constant *k*_1_ decreased from 0.28 to 0.22 1/min, the Holman parameter *k*_*H*_ decreased from 35.8 to 27.5%/√min, and the index *n* decreased from 0.35 to 0.30, indicating the predominance of the diffusion mechanism of release. These results are consistent with the Higuchi and Korsmeyer–Peppas models. The initial release stage is determined by the rapid release of the API from the matrix’s surface and weakly bound sites, both of which are sensitive to the degree of compaction. The subsequent stage, however, is governed by diffusion within the denser structure. Thus, an increase in pressure during compression leads to an increase in density and a decrease in the porosity of the matrix, which limits solvent penetration and API diffusion, thereby slowing down the release and confirming the patterns identified both through kinetic parameters and through the fitting of experimental data to kinetic models.

Thus, analysis of the experimental data indicates that the use of lower compression forces is advisable in view of the formation of a better texture and favourable MCG release profiles. At the same time, increasing the compression force to 7 kN improves the mechanical stability of the samples by reducing their friability compared to 5 kN, without significantly affecting the initial hardness and the kinetics of APIs release from the MCG. Based on these results obtained, a compression force of 7 kN was selected as optimal for further use in the manufacturing process of the developed chewing gums.

Once the optimal compression force has been determined, it is advisable to conduct preclinical pharmacological studies of the formulation, given the potential effect of its components—lysozyme hydrochloride and ascorbic acid—on the symptoms of xerostomia. This justifies the need to assess the effect of the APIs on salivation processes and the functional state of the salivary glands under conditions of experimental atropine-induced xerostomia.

**Figure 11:**
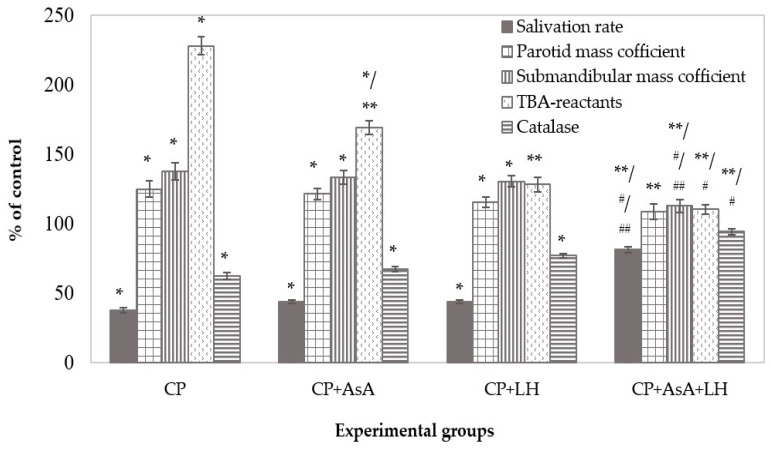
Integrated effects on salivary gland function and oxidative stress markers (the intact control group was taken as 100%; *n* = 8 per group, values are expressed as average ± SD); *—differences are significant relative to the IC group values, Tukey HSD test, *p* < 0.05; **—differences are significant relative to the CP group values, Tukey HSD test, *p* < 0.05; #—differences are significant relative to the CP + AsA group values, Tukey HSD test, *p* < 0.05; ##—differences are significant relative to the CP + LH group values, Tukey HSD test, *p* < 0.05.

In the study, lysozyme hydrochloride and ascorbic acid were administered separately and in combination to assess individual and joint effects. The compositions were administered by irrigating the oral cavity with an aliquot collected after a chewing gum release test; according to the control parameters, this method of administration was equivalent to using of chewing gum. During the investigation, the following parameters were assessed: spontaneous salivation rate, mass coefficients of the parotid and submandibular salivary glands (as the principal exocrine glands responsible for saliva production in rodents and commonly used target organs in experimental xerostomia models [97,98], and indicators of oxidative stress associated with xerostomia pathogenesis. Oxidative stress was evaluated due to its established role in salivary gland dysfunction, where excessive production of reactive oxygen species and impairment of antioxidant defense systems contribute to reduced salivary secretion and tissue damage [28,99]. Therefore, lipid peroxidation and antioxidant defense status were evaluated using TBA-reactive substances as an indicator of oxidative damage and catalase activity as a key enzyme of the endogenous antioxidant defense system [28,100]. To standardize the data and enable a comparative analysis of indicators expressed in different units of measurement, the results are presented as a percentage of the control group, taken as 100%. The summary results are shown in Figure 11.

Analysis of the data obtained indicates that, under conditions of experimental pathology, there is a reduction in salivary flow rates and changes in the functional state of the salivary glands compared with the intact control group. Following daily irrigation of the oral cavity with atropine sulphate in experimental animals, a statistically significant increase in the mass coefficients of the parotid and submandibular salivary glands was observed. According to the literature, this effect is due to the narrowing of the salivary ducts, the accumulation of saliva and the development of a local inflammatory process in the salivary glands [70]. The individual administration of ascorbic acid (CP + AsA group) or lysozyme hydrochloride (CP + LH group) contributes to the partial normalization of certain parameters. However, no statistically significant changes were observed in salivary flow rate or salivary gland mass coefficients compared with the control pathology group (CP). In contrast, their combination (group CP + AsA + LH) resulted in a statistically significant increase in saliva volume, which rose 2.17-fold compared with the CP group and approached the values of the intact control. Furthermore, the combined use of the APIs resulted in a statistically significant reduction in the mass coefficients of the parotid (by 13.0%) and submandibular salivary glands (by 18.1%) compared with the control group, as well as compared with their individual use (CP + AsA and CP + LH), indicating a potentiating cumulative effect of the components under investigation.

A marked imbalance in the pro-oxidant/antioxidant system was observed in the homogenate of the submandibular salivary glands of rats with experimental xerostomia: an increase in TBA reactant levels and a decrease in catalase activity, indicating the activation of free-radical processes. Using ascorbic acid alone reduced the level of TBA-reactants by 25.8%, while lysozyme hydrochloride reduced it by 43.7% with no significant effect on catalase activity. In contrast, combining the two APIs ensured almost complete normalization of antioxidant status: TBA-reactant levels decreased by 51.6% and catalase activity increased by 51.0%, compared to the control group.

Thus, the obtained data demonstrate that the combination of ascorbic acid and lysozyme hydrochloride in the medicated chewing gum contributed to restoration of salivary secretion and improvement of morphofunctional and antioxidant parameters of salivary glands under conditions of atropine-induced xerostomia. These findings indicate the potential of the investigated combination for correction of xerostomia-associated salivary dysfunction.

The obtained results are consistent with previously published experimental studies on xerostomia and salivary gland dysfunction.

Westlind-Danielsson et al., in studies conducted on rats, demonstrated that atropine-induced blockade of muscarinic cholinergic signaling leads to suppression of salivary secretion and functional alterations of salivary glands [69]. These findings are in line with the present results obtained in the atropine-induced xerostomia model, where reduced salivary flow and changes in salivary gland parameters were also observed under experimental conditions.

Kim Y.-J. and co-authors established that oxidative stress is one of the key mechanisms involved in xerostomia pathogenesis, where excessive accumulation of reactive oxygen species and impairment of antioxidant defense systems contribute to salivary gland dysfunction and decreased salivary secretion [28]. Similar alterations in oxidative balance were observed in the present study, as evidenced by increased TBA-reactive substances and decreased catalase activity in salivary gland tissues.

Studies by Toan N.K. et al., performed in experimental animal models, demonstrated that ascorbic acid improves salivary gland function and stimulates salivary secretion through modulation of cholinergic signaling and restoration of glandular activity [101]. In addition, Tenovuo reported that lysozyme is an important component of the innate salivary defense system, contributing to maintenance of oral homeostasis under xerostomic conditions due to its antimicrobial and protective properties [102]. Kirstilä et al. also showed that lysozyme-containing formulations may alleviate xerostomia symptoms and improve salivary protective functions [103]. These literature data are in agreement with the present findings, where combined administration of ascorbic acid and lysozyme hydrochloride resulted in improved salivary secretion and normalization of morphofunctional and antioxidant parameters in rats with atropine-induced xerostomia.

Despite these findings, several limitations of the present study should be acknowledged.

The atropine-induced xerostomia model represents a well-established pharmacological approach for transient suppression of salivary secretion via competitive muscarinic receptor antagonism, primarily affecting M3-mediated parasympathetic regulation of salivary glands [12]. It is particularly suitable for evaluating acute secretagogue activity and mechanistic effects under controlled experimental conditions. However, it does not fully reproduce the chronic and multifactorial nature of clinical xerostomia, such as that observed in Sjögren’s syndrome, head and neck radiotherapy, or long-term polypharmacy, as it primarily induces a reversible receptor-mediated inhibition without structural or inflammatory gland alterations [28,99]. Despite these limitations, the model provides a robust and reproducible platform for preclinical screening of salivation-modulating compounds [97].

Rodent models are widely utilized in salivary gland research due to their well-characterized autonomic regulation and practical advantages for controlled experimental design. Rats in particular enable repeated sampling and invasive physiological assessments that are not feasible in human studies [97]. However, translational limitations must be considered. Species differences in salivary gland anatomy, receptor distribution, and saliva composition may influence pharmacodynamic responses and limit direct extrapolation to humans [98]. Moreover, xerostomia in humans is a subjective and multifactorial condition, encompassing not only reduced salivary flow but also alterations in saliva composition and oral mucosal perception, which cannot be fully replicated in rodent models [12,100]. Nevertheless, the rat model remains highly valuable for preclinical assessment of pharmacological effects on salivary secretion. When interpreted within its methodological constraints and complemented by clinical evidence, it provides relevant mechanistic insights and supports early-stage translational research in xerostomia-related therapies.

A potential limitation of the present study is also the use of thiopental anaesthesia, which may have influenced salivary secretion. Thiopental exerts central nervous system depressant effects, potentially reducing autonomic outflow and parasympathetic stimulation of salivary glands, leading to a transient decrease in salivary flow [104]. In addition, anaesthesia may affect haemodynamic parameters and glandular perfusion, further contributing to variability in salivary measurements [105]. Although these effects cannot be fully excluded, a standardized anaesthetic protocol across all experimental groups ensured consistency of conditions and minimized inter-group variability, thereby supporting the reliability of the comparative analysis.

Additionally, a limitation of the present study is the use of a limited set of oxidative stress biomarkers, namely TBA-reactive substances and catalase activity, while other relevant parameters of oxidative stress and antioxidant defense were not assessed. Inclusion of these parameters in future studies may provide a more comprehensive understanding of the oxidative stress-related mechanisms involved in xerostomia and the effects of the investigated formulation.

An additional limitation of the present study is that the *in vivo* experiments did not replicate the mechanical mastication process characteristic of medicated chewing gum use in humans. In the animal model, the formulation was administered as an aqueous aliquot following *in vitro* release testing, which does not fully reproduce physiological conditions of chewing, including mechanical stimulation of salivary glands and increased salivary flow. It is well established that mastication itself is a key physiological stimulus of salivary secretion and can significantly enhance salivary flow rates, thereby contributing to the overall effectiveness of chewing gum-based interventions in xerostomia management [100]. Clinical and systematic evidence further confirms that sugar-free chewing gum may increase salivary secretion and alleviate symptoms of dry mouth in affected patients [21,33]. Therefore, future studies in human subjects are required to evaluate the combined mechanical and pharmacological effects of the developed medicated chewing gum system under real-use conditions.

Nevertheless, the present study provides insights into salivary gland responses under experimental xerostomia conditions and highlights the combined effects of ascorbic acid and lysozyme hydrochloride in the developed medicated chewing gum formulation.

## 4. Conclusions

The present study reports the development and experimental evaluation of a novel compressed medicated chewing gum containing lysozyme hydrochloride and ascorbic acid for xerostomia-related salivary dysfunction.

Evaluation of the physicochemical and technological properties of the active ingredients and their mixtures with excipients enabled the selection of appropriate incorporation methods into the compression mass. In particular, lysozyme hydrochloride was subjected to preliminary wet granulation to improve its technological performance during compression, whereas ascorbic acid was introduced via premixing to minimize oxidative degradation. In addition, reduced relative humidity was identified as an important factor for ensuring process stability due to the hygroscopic nature of the formulation components. Optimization of compression parameters ensured adequate mechanical properties and rapid release of the active ingredients under experimental conditions.

The technological approaches applied in this study may facilitate the development of similar compressed medicated chewing gum formulations containing other active pharmaceutical ingredients.

Preclinical results demonstrated that the combination of lysozyme hydrochloride and ascorbic acid produced a more pronounced corrective effect in rats with atropine-induced xerostomia compared with individual components. The combined formulation restored salivary secretion, normalized morphofunctional parameters of the salivary glands, and improved antioxidant status under experimental conditions, whereas individual substances provided only partial correction of the evaluated parameters.

Overall, the findings indicate the potential applicability of the developed formulation for xerostomia-related salivary dysfunction and oral health support. Nevertheless, additional studies in clinically relevant models and human subjects are required to confirm its translational relevance, mechanisms of action, and clinical efficacy.

## Figures and Tables

**Table 1:** Kinetic models and their corresponding equations, used to analyze drug release.

Model	Equation	Description
Zero-order	$Q_{t}=Q_{0}+k_{0}t$	Constant drug release rate
First-order	${ln(100-Q}_{t})=\ln100\;-\;k_{1}t$	Concentration-dependent release
Higuchi	$Q_{t}=k_{H}\sqrt{t}$	Diffusion-controlled release
Korsmeyer–Peppas	$\frac{M_{t}}{M_{\infty }}=kt^{n}$	Mechanism of release

Note: *Qₜ* is the amount of drug released at time *t*, *Q*_0_ is the initial amount of drug, *k*_0_, *k*_1_, and *k_H_* are release rate constants, *Mₜ/M_∞_* is the fraction of drug released, *k* is the kinetic constant, and *n* is the release exponent indicating the mechanism of drug release.

**Table 2:** Selection of the composition of the compressed MCGs under development: components, its role and critical parameters.

Component	Functional Purpose	Critical Parameters
Ascorbic acid (AsA)	API: antioxidant protection; anti-inflammatory, immunomodulatory and regenerative effects; stimulation of saliva secretion	stability to moisture and temperature; susceptibility to oxidation; taste; safe concentration; uniformity of distribution throughout the mixture
Lysozyme hydrochloride (LH)	API: antimicrobial, anti-inflammatory, reparative, immunostimulatory and antiviral activity; prevention of microbial adhesion to teeth	preservation of biological activity; sensitivity to temperature and moisture; uniform distribution throughout the mass
Composition HiG PWD-01	Chewable base: carrier matrix; development of chewable texture and elasticity; control of active ingredient release	textural properties (plasticity, elasticity); sensitivity to temperature and moisture; ability to retain powders and liquids
Sucralose	Intensive sweetener: improved flavour; masking of acidity; increased compliance	intensity of sweetness; absence of a bitter (unpleasant) aftertaste; non-cariogenic properties; uniform distribution throughout the mixture
Powdered flavouring agent “Green Apple”	Flavour enhancer: rapid development of the flavour profile	taste and absence of bitterness; particle size; uniformity of distribution throughout the mixture
Aroma flavouring “Green Apple”	Flavour and aroma enhancer: creates a lingering taste and aroma when chewed	resistance to oxidation; method of incorporation into the powder mixture and uniformity of distribution within it
Aerosil 380; Syloid^®^ 244FP; Neusilin^®^ ULP2	Adsorbent/glidant: adsorption of liquid flavourings and their conversion into powder form; improvement of flow properties; prevention of agglomeration	adsorption capacity; specific surface area; effect on mixture flowability; effect on flavour release and API
Magnesium stearate	Lubricant: lubrication; reduction in friction; prevention of sticking to equipment	distribution within the mixture; effect on compressibility and moulding; potential effect on the release of API

**Table 3:** Technological properties of the APIs and HiG^®^ PWD-01 chewable base (*n* = 3, *p* < 0.05, values are expressed as average ± SD).

Technological Indicators	Results		
	AsA	LH	HiG^®^ PWD-01
Flowability, s/100 g of sample:			
stationary funnel	7.90 ± 1.09	∞	7.45 ± 0.38
with vibrating funnel		22.46 ± 5.22	
The angle of repose: tan(α), deg.	35.75 ± 0.74	–	29.26 ± 1.61
protractor, deg.	37.60 ± 0.68	42.80 ± 1.62	30.00 ± 2.91
Bulk volume, V_o_, mL	167.1 ± 0.9	170.6 ± 0.5	155.9 ± 0.3
Tapped volume, V_1250_, mL	134.5 ± 1.4	129.4 ± 0.5	135.8 ± 0.3
Bulk density, m/V_o_, g/mL	0.902 ± 0.013	0.586 ± 0.002	0.642 ± 0.001
Tapped density, m/V_1250_, g/mL	1.120 ± 0.013	0.773 ± 0.003	0.737 ± 0.002
Carr’s index, %	19	24	13
Hausner ratio	1.24	1.32	1.15

**Table 4:** Technological properties of masses for pressing I and II (*n* = 3, *p* < 0.05, values are expressed as average ± SD).

Technological Indicators	Results	
	Mass I (Physical Mixture)	Mass II (Pre-Granulation of LH)
Flowability, s/100 g of sample	8.95 ± 0.58	7.43 ± 0.65
The angle of repose: tan(α), deg.	37.83 ± 1.02	30.65 ± 1.70
protractor, deg.	36.65 ± 2.12	31.25 ± 2.72
Bulk volume, V_o_, mL	146.1 ± 0.5	154.3 ± 0.6
Tapped volume, V_1250_, mL	121.6 ± 0.3	135.0 ± 0.4
Bulk density, m/V_o_, g/mL	0.705 ± 0.007	0.648 ± 0.003
Tapped density, m/V_1250_, g/mL	0.847 ± 0.009	0.740 ± 0.002
Carr’s index, %	17	13
Hausner ratio	1.20	1.14

**Table 5:** Technological properties of the mass for pressing with studied adsorbents (*n* = 3, values are expressed as average ± SD).

Technological Indicators	Results	
	with Syloid^®^ 244FP	with Neusilin^®^ ULP2
Flowability, s/100 g of sample	6.13 ± 0.21 *	6.59 ± 0.09
The angle of repose: tan(α), deg.	29.76 ± 1.01	30.25 ± 0.54
protractor, deg.	31.00 ± 2.91	32.40 ± 0.48
Bulk volume, V_o_, mL	147.9 ± 0.5	147.7 ± 0.9
Tapped volume, V_1250_, mL	126.3 ± 0.7	128.5 ± 0.4
Bulk density, m/V_o_, g/mL	0.682 ± 0.002	0.677 ± 0.003
Tapped density, m/V_1250_, g/mL	0.790 ± 0.004	0.798 ± 0.003
Carr’s index, %	14	15
Hausner ratio	1.16	1.18

Note: *—statistically significant difference compared to the Neusilin^®^ ULP2 batch (*p* < 0.05, Student’s *t*-test).

**Table 6:** Technological properties of the final MCG mass for pressing (*n* = 3, *p* < 0.05, values are expressed as average ± SD).

Technological Indicators	Results
Flowability, s/100 g of sample	5.94 ± 0.25
The angle of repose: tan(α), deg.	27.65 ± 0.87
protractor, deg.	28.33 ± 1.03
Bulk volume, V_o_, mL	143.1 ± 0.2
Tapped volume, V_1250_, mL	131.0 ± 0.3
Bulk density, m/V_o_, g/mL	0.699 ± 0.005
Tapped density, m/V_1250_, g/mL	0.763 ± 0.004
Carr’s index, %	8
Hausner ratio	1.09

**Table 7:** Geometric parameters and strength characteristics of MCG (*n* = 3, values are expressed as average ± SD).

Compression Force, kN	Geometric Parameters, mm		Resistance to Crushing, N	Friability, %	Penetration Test	
	Diameter	Thickness			Hardness, g	Adhesion, g·sec
5	13.02 ± 0.01	5.62 ± 0.03	71 ± 2	0.121 ± 0.001	2429.70 ± 80.37	11.68 ± 3.12
7	13.01 ± 0.01	5.50 ± 0.02	78 ± 2	0.102 ± 0.001	2589.50 ± 91.48	14.31 ± 3.37
10	13.01 ± 0.01	5.44 ± 0.05	80 ± 1	0.090 ± 0.001	2977.08 ± 163.73	19.71 ± 2.11
15	13.00 ± 0.01	5.21 ± 0.03	92 ± 2	0.054 ± 0.001	4001.93 ± 186.76	28.78 ± 4.99

Note: Statistical analysis was performed using one-way analysis of variance (ANOVA). Pairwise comparisons indicated that the differences between the batches (5, 7, 10, and 15 kN) are statistically significant (*p* < 0.05) for all indicators except the diameter.

**Table 8:** Parameters of API release from MCG at different compression forces, determined using kinetic models.

Pressure (kN)	*k*_0_ (%/min)	*k*_1_ (1/min)	*k_H_* (%/√min)	*n* (Korsmeyer–Peppas)
5	3.00 ± 0.15	0.28 ± 0.01	35.80 ± 1.10	0.35 ± 0.02
7	2.80 ± 0.12	0.27 ± 0.01	33.50 ± 0.90	0.33 ± 0.02
10	2.40 ± 0.10	0.25 ± 0.01	30.10 ± 1.00	0.31 ± 0.01
15	2.00 ± 0.08	0.22 ± 0.01	27.50 ± 0.80	0.30 ± 0.01

Note: values are expressed as average ± SD. *k*_0_—initial release rate (Zero/First-order, %/min), *k*_1_—rate constant (1/min), *k_H_*—Higuchi parameter (Higuchi, %/√min), *n*—parameter characterizing the release mechanism (Korsmeyer–Peppas).

## Data Availability

The original contributions presented in this study are included in the article. Further inquiries can be directed to the corresponding author.
